# Ethanol at Subinhibitory Concentrations Enhances Biofilm Formation in *Salmonella* Enteritidis

**DOI:** 10.3390/foods11152237

**Published:** 2022-07-27

**Authors:** Shoukui He, Zeqiang Zhan, Chunlei Shi, Siyun Wang, Xianming Shi

**Affiliations:** 1 MOST-USDA Joint Research Center for Food Safety, School of Agriculture and Biology, State Key Laboratory of Microbial Metabolism, Shanghai Jiao Tong University, Shanghai 200240, China; heshoukui@sjtu.edu.cn (S.H.); zhanzeqiang@sjtu.edu.cn (Z.Z.); clshi@sjtu.edu.cn (C.S.); 2 Food, Nutrition and Health, Faculty of Land and Food Systems, The University of British Columbia, Vancouver, BC V6T 1Z4, Canada; siyun.wang@ubc.ca

**Keywords:** *Salmonella* Enteritidis, biofilm formation, ethanol stress, cell characteristic, gene expression, quorum sensing

## Abstract

The survival of *Salmonella* Enteritidis in the food chain is relevant to its biofilm formation capacity, which is influenced by suboptimal environmental conditions. Here, biofilm formation pattern of this bacterium was assessed in the presence of ethanol at sub-minimal inhibitory concentrations (sub-MICs) by microtiter plate assays, cell characteristic analyses, and gene expression tests. It was observed that ethanol at subinhibitory concentrations (1/4 MIC, 2.5%; 1/2 MIC, 5.0%) was able to stimulate biofilm formation in *S.* Enteritidis. The OD_595_ value (optical density at 595 nm) used to quantify biofilm production was increased from 0.14 in control groups to 0.36 and 0.63 under 2.5% and 5.0% ethanol stresses, respectively. Ethanol was also shown to reduce bacterial swimming motility and enhance cell auto-aggregation ability. However, other cell characteristics such as swarming activity, initial attachment and cell surface hydrophobicity were not remarkedly impacted by ethanol. Reverse transcription quantitative real-time PCR (RT-qPCR) analysis further revealed that the *luxS* gene belonging to a quorum-sensing system was upregulated by 2.49- and 10.08-fold in the presence of 2.5% and 5.0% ethanol, respectively. The relative expression level of other biofilm-related genes (*adrA*, *csgB*, *csgD*, and *sdiA*) and sRNAs (ArcZ, CsrB, OxyS, and SroC) did not obviously change. Taken together, these findings suggest that decrease in swimming motility and increase in cell auto-aggregation and quorum sensing may result in the enhancement of biofilm formation by *S.* Enteritidis under sublethal ethanol stress.

## 1. Introduction

Ethanol is a common component present in a variety of foods such as alcoholic beverages, fruit products, and baked products [1,2,3]. For a long time, ethanol has been utilized as a chemical disinfectant for food contact surfaces, conveyor belts, as well as food processing tools [4,5,6]. Moreover, ethanol is effective for controlling postharvest decay and extending shelf life in the fruit industry [7]. Occasionally, ethanol can be present in food-related environments at subinhibitory concentrations due to its easy evaporation, inappropriate application, or dilution in the environment. The subinhibitory concentrations of ethanol are able to exert an inhibitory activity but are not lethal to bacterial pathogens [6].

Ethanol at subinhibitory concentrations has been recognized as a crucial environmental factor influencing biofilm formation of bacterial pathogens. Tango et al. [8] found that 2.5–3.5% ethanol enabled *Staphylococcus aureus* ATCC 13150 to form more biofilms. Similarly, biofilm formation in *Pseudomonas aeruginosa* PAO1 was also induced by ethanol at low levels (1% and 2%) [9]. Bacterial pathogens in biofilms are generally resistant to cleaning and sanitation operations and are extremely difficult to eradicate in food industries [10,11]. In this context, ethanol-triggered biofilm formation can contribute to the survival and persistence of bacterial pathogens on industrial surfaces and equipment, thus posing a threat to food safety. It is thus essential to reveal biofilm formation patterns of other important bacterial pathogens such as *Salmonella* spp. under ethanol stress.

Biofilm formation mechanisms of *Salmonella* spp. have been well characterized in laboratory conditions that are optimal for bacterial growth [12]. In terms of physiological strategies, cell surface characteristics (e.g., motility, hydrophobicity, and auto-aggregation) have been demonstrated to participate in biofilm production of this bacterium [13]. At the molecular level, attachment genes (e.g., *adrA, csgB*, and *csgD*) and quorum-sensing genes (e.g., *luxS* and *sdiA*) have been recognized as key genetic elements for biofilm formation [14]. More recently, a couple of small RNAs (sRNAs) (e.g., ArcZ, CsrB, OxyS, and SroC) have also been revealed to modulate biofilm formation of *Salmonella* spp. [15]. Nevertheless, it remains unknown whether the aforementioned mechanisms function in *Salmonella* spp. biofilm formation under ethanol stress.

Practically, the mode of action of biofilm formation by *Salmonella* spp. under several environmental stress conditions has been characterized in previous studies [16,17]. For instance, *Salmonella* Typhimurium displayed reduced biofilm formation when exposed to sub-MIC levels of lactobionic acid, which was probably due to repressed synthesis of extracellular polymeric substances, reduced ability of cell motilities, and decreased expression of genes (e.g., *adrA*, *flhD*, and *fljB*) and sRNAs (e.g., ArcZ, CsrB, and SroC) [16]. In addition, the expression of quorum-sensing genes (e.g., *luxS*) and stress response genes (e.g., *rpoS*) was involved in biofilm formation of *S*. Enteritidis and *S*. Typhimurium in response to quercetin [17]. Thus, these kinds of physiological and gene expression analyses will be helpful in uncovering mechanisms of bacterial biofilm formation under other stress conditions such as exposure to alcoholic disinfectants.

The current work was carried out to evaluate the influence of ethanol at subinhibitory concentrations on biofilm formation in *S*. Enteritidis, a leading serotype of *Salmonella* spp. responsible for foodborne diseases. The underlying mechanisms on biofilm formation of this pathogen under ethanol stress will also be investigated via analysis of cell surface characteristics and gene expression levels.

## 2. Materials and Methods

### 2.1. Target Bacteria

*S.* Enteritidis ATCC 13076 was kept in Luria-Bertani (LB) broth containing 50% glycerol at −80 °C. This strain was resuscitated by inoculating stock culture onto LB agar and incubating, at 37 °C, for 24 h. A single bacterial colony was then transferred into LB broth, followed by overnight incubation, at 37 °C, before each test.

### 2.2. Antibacterial Activity Test

The two-fold dilution method was employed to assess the inhibitory activity of ethanol on *S.* Enteritidis [18]. An aliquot (5 μL) of an overnight culture (approximately 10^9^ CFU/mL) was added into 5 mL TSB-YE (tryptic soy broth supplemented with 0.6% yeast extract) containing ethanol ranging from 2.5% to 40% (*v*/*v*). Negative (5 mL TSB-YE) and positive (5 mL TSB-YE containing 5 μL bacterial suspensions) controls were also included. The samples were stored at 25 °C (room temperature) on a rotator (200 rpm) for 24 h. This temperature was selected because it might be encountered by bacterial pathogens in actual conditions during food processing [19] and temperature abuse scenarios during food storage [20]. Optical density at 600 nm was monitored to measure bacterial growth in different concentrations of ethanol. The minimum inhibitory concentration (MIC) was recorded as the lowest ethanol concentration that completely retarded bacterial growth.

### 2.3. Biofilm Formation Assay

Biofilm production of *S.* Enteritidis in the presence of sub-MIC levels (1/4 MIC and 1/2 MIC) of ethanol was quantified using the 96-well microtiter plate assay [21]. The overnight bacteria culture was diluted to an inoculum level of approximately 10^6^ CFU/mL with TSB-YE, and ethanol was later added to achieve a final concentration corresponding to 0, 1/4, and 1/2 of the MIC, respectively. The prepared samples were dispensed into 96-well plates with a volume of 200 μL, followed by static incubation at 25 °C for 72 h. After the biofilm formation, the inoculum was aspirated from 96-well plates, and sterile distilled water was then utilized to wash each well three times. The plates were air-dried at 25 °C for 15 min, and 0.1% (*w*/*v*) crystal violet solution (200 μL) was transferred into each well. After dyeing for 45 min, crystal violet was discarded from each well. The plates were allowed to air dry after washing with sterile distilled water, and 95% (*v*/*v*) ethanol (200 μL) was then added to each well. After gentle vortex mixing for 45 min, biofilm formation in each well was quantified by optical density at 595 nm (OD_595_).

### 2.4. Bacterial Attachment Assay

Bacterial adhesion to polystyrene surfaces was measured as previously detailed by Nilsson et al. [22] and do Valle Gomes et al. [23]. The 24-well microtiter plates (Falcon, Franklin Lakes, NJ, USA) were utilized to increase surface binding areas. The overnight culture of *S.* Enteritidis was diluted to approximately 10^6^ CFU/mL with TSB-YE and supplemented with 0, 1/4, and 1/2 MIC of ethanol, respectively. An aliquot (3 mL) of the resulting suspensions was then transferred to 24-well plates. After static incubation at 25 °C for 5 h, cell suspensions were discarded from each well, followed by washing with sterile distilled water. Subsequently, the wells were stained with crystal violet (0.1%, *w*/*v*), and added with ethanol (95%, *v*/*v*). The attachment ability of *S.* Enteritidis was estimated by reading OD_595_ values (Tecan Sunrise, Tecan Group Ltd., Mannedorf, Switzerland).

### 2.5. Cell Motility Test

Cell motility test was conducted based on the method detailed by Roy et al. [24]. The medium was prepared by adding 0.3% and 0.6% agar to TSB-YE for the swimming and swarming assays, respectively, followed by autoclaving. Ethanol was then added to each medium before it hardened and mixed thoroughly. The final concentration of ethanol was 0, 1/4, and 1/2 MIC, respectively. In the swimming assay, 2 μL of pre-diluted bacterial suspension (approximately 10^6^ CFU/mL) was inoculated by passing through a fresh 0.3% agar plate. An aliquot of 5 μL pre-diluted bacterial suspension (approximately 10^6^ CFU/mL) was spotted onto the center of a fresh 0.6% agar plate in the swarming assay. The plates were then stored at 25 °C for 10 and 24 h, respectively. The motility zone diameter was recorded as the distance between the center of the plate and the leading edge of bacterial growth.

### 2.6. Cell Surface Hydrophobicity Study

Bacterial cell surface hydrophobicity was estimated by a microbial adhesion to solvents test [25,26]. *S*. Enteritidis cells cultured as described in Section 2.3 were spun, washed, and redissolved in phosphate-buffered saline. The optical density of the resulting bacterial suspensions was recorded at 600 nm (OD_600_ pre-vortex). Afterwards, 3 mL of hexadecane was added to an equal volume of cell suspensions. Each bacterial suspension was vortexed and incubated for 15 min at 25 °C. The aqueous phase was then subjected to optical density quantification at 600 nm (OD_600_ post-vortex). Bacterial cell surface hydrophobicity was estimated by the following formula: hydrophobicity (%) = (1 − OD_600_ post-vortex/OD_600_ pre-vortex) × 100.

### 2.7. Cell Auto-Aggregation Assay

The cell auto-aggregation property was estimated as previously detailed by Lee et al. [27] with slight modifications. *S*. Enteritidis cells cultured as described in Section 2.3 were centrifuged and resuspended in an equal volume of TSB-YE. An aliquot (200 μL) of bacterial suspensions was mixed with 4.8 mL fresh TSB-YE. After overnight incubation at 25 °C statically, the optical density at 600 nm (OD_600_ pre-vortex) of the upper layer was checked. The samples were then vortexed carefully and evaluated at 600 nm (OD_600_ post-vortex). Bacterial cell auto-aggregation was calculated using the following formula: auto-aggregation (%) = (1 − OD_600_ pre-vortex/OD_600_ post-vortex) × 100.

### 2.8. Gene Expression Analysis

The RT-qPCR test was carried out to quantify gene expression levels [28,29]. The TRIzol™ reagent (Invitrogen, Carlsbad, CA, USA) was utilized to extract total RNA from *S*. Enteritidis cells cultured as described in Section 2.3. The cDNA was then synthesized using a HiScript RT SuperMix for qPCR (+gDNA wiper) Kit (Vazyme Biotech Co., Ltd., Nanjing, China). Primers in Table 1 were referenced from published data [14,15]. PCR amplification was initiated at 95 °C for 5 min, followed by 40 cycles at 95 °C for 5 s, 55 °C for 15 s, and 68 °C for 30 s. Change in gene expression levels of *S*. Enteritidis under ethanol stress was compared with that in pure TSB-YE with 16S rRNA as the reference gene by the 2^−ΔΔCt^ method [30].

### 2.9. Statistical Analysis

The results from at least three biological experiments were presented as means ± standard deviations. Data were submitted to a one-way Duncan′s ANOVA analysis in the SAS program at the level of *p* < 0.05.

## 3. Results and Discussion

### 3.1. Influence of Ethanol at Subinhibitory Concentrations on Bacterial Biofilm Formation

**Figure 1:**
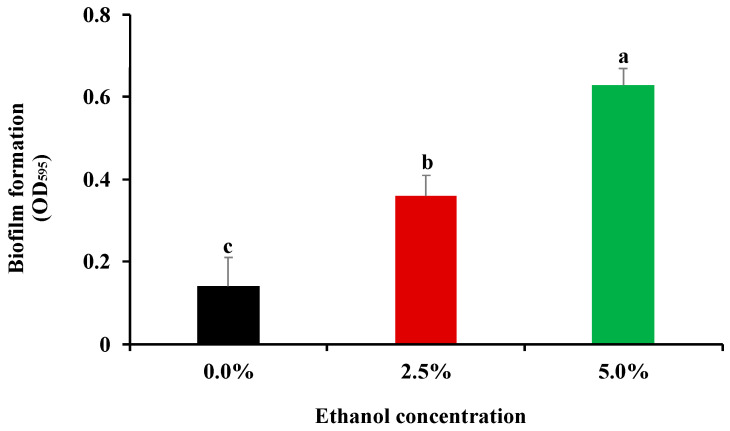
Influence of subinhibitory concentrations of ethanol (1/4 MIC, 2.5%; 1/2 MIC, 5.0%) on biofilm formation of *S*. Enteritidis. Different letters above vertical bars represent a significant difference (*p* < 0.05).

Biofilm formation of *Salmonella* spp. under suboptimal environmental conditions such as acidic pH and high/low temperature has been revealed in previous studies [31,32]. Nevertheless, the capacity of this bacterial pathogen to form biofilms in response to alcoholic disinfectants remains largely unknown. Therefore, the inhibitory activity of ethanol on *S*. Enteritidis was initially evaluated to screen appropriate concentrations of ethanol for subsequent biofilm formation assay in the current work. The MIC value of ethanol was found to be 10% against *S*. Enteritidis. Sub-MIC levels of antimicrobials have been considered to exert an inhibitory but not lethal activity against bacterial pathogens [18,33]. Hence, 2.5% (1/4 MIC) and 5.0% (1/2 MIC) were selected as subinhibitory ethanol concentrations for subsequent biofilm formation assay. As shown in Figure 1, exposure to ethanol at subinhibitory levels (2.5% and 5.0%) significantly (*p* < 0.05) increased biofilm production ability of *S*. Enteritidis. Moreover, a higher amount of biofilm was produced when ethanol concentration was increased from 2.5% to 5.0%. These results suggest that sublethal ethanol stress stimulated biofilm formation in *S*. Enteritidis.

Ethanol-mediated biofilm formation has also been assessed in other bacterial pathogens. Tashiro et al. [9] reported that ethanol at low concentrations (1% and 2%) triggered biofilm production by *P. aeruginosa*. Moreover, biofilm formation ability of *S. aureus* in tryptic soy broth (TSB) containing 2–8% ethanol was also greater than that in TSB alone [34]. Biofilm growth can provide pathogens with an evaluated degree of resistance to physical and chemical agents commonly applied during food processing, thus serving as a vital source of bacterial contamination in food industries [8]. Therefore, ethanol at low concentrations poses a potential threat to food safety considering its ability to stimulate bacterial biofilm formation.

### 3.2. Influence of Ethanol at Subinhibitory Concentrations on Cell Attachment Ability

**Figure 2:**
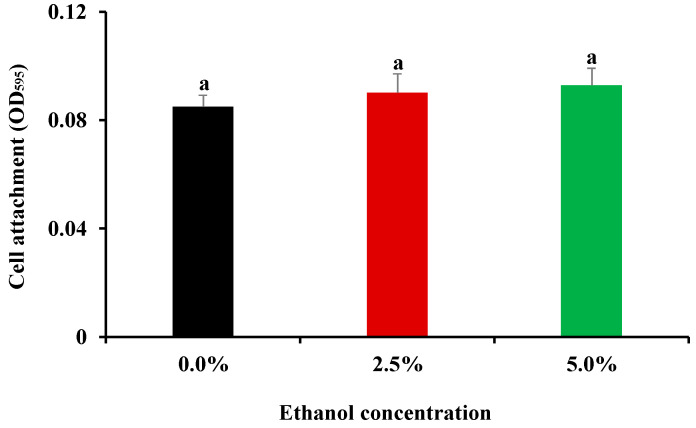
Influence of subinhibitory concentrations of ethanol (1/4 MIC, 2.5%; 1/2 MIC, 5.0%) on cell attachment of *S*. Enteritidis. The same letters above vertical bars represent no significant difference (*p* > 0.05).

The influence of ethanol at subinhibitory concentrations on bacterial attachment is presented in Figure 2. No significant (*p* > 0.05) difference in the initial attachment ability was observed among *S*. Enteritidis cells grown in pure TSB-YE or in TSB-YE supplemented with 2.5% and 5.0% ethanol. Actually, cell adherence to the surface of polystyrene and other substances is the initial step for bacterial pathogens to produce biofilms [35,36]. It was thus indicative that ethanol at subinhibitory levels might not contribute to biofilm formation in *S*. Enteritidis by affecting its initial attachment in the current work.

### 3.3. Influence of Ethanol at Subinhibitory Concentrations on Bacterial Motility Capacity

**Figure 3:**
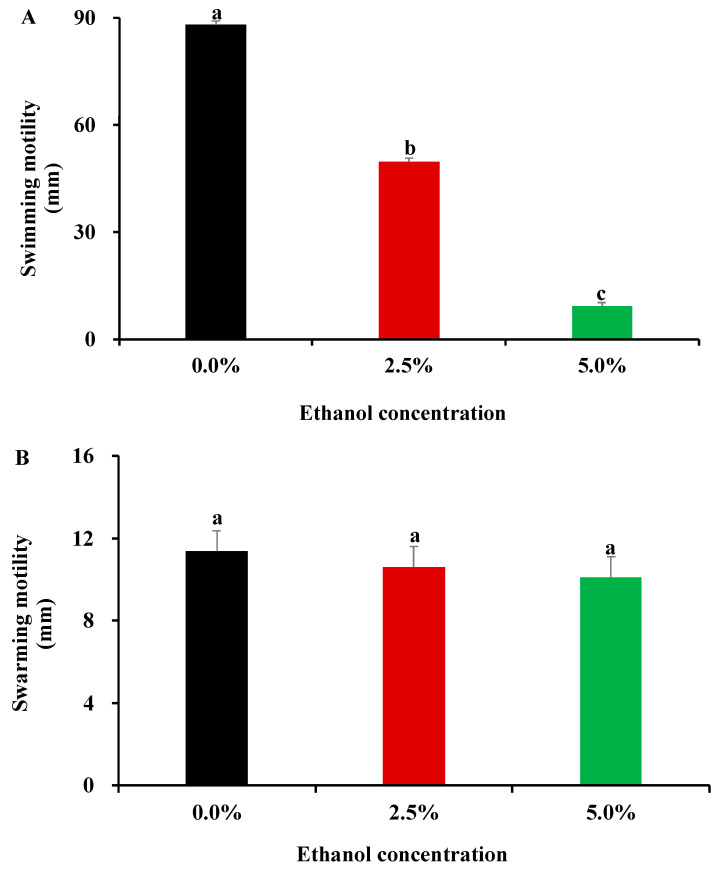
Influence of subinhibitory concentrations of ethanol (1/4 MIC, 2.5%; 1/2 MIC, 5.0%) on cell motility of *S*. Enteritidis. (**A**) Swimming motility; (**B**) Swarming motility. Different letters above vertical bars represent a significant difference (*p* < 0.05).

Bacterial mobility in the presence of ethanol was determined by swimming and swarming assays in the current work. In the swimming assay, *S*. Enteritidis motility on 0.3% soft-agar plates was partially and completely inhibited by 2.5% and 5.0% ethanol, respectively (Figure 3A). Nevertheless, *S*. Enteritidis did not alter its swarming ability on 0.6% soft-agar plates under ethanol stress (Figure 3B). Therefore, ethanol at subinhibitory concentrations exerted an inhibitory effect on swimming motility, but not on swarming ability in *S*. Enteritidis.

Swimming motility is a type of bacterial behavior on semisolid agar media propelled by flagella, which is proven instrumental in biofilm formation [37]. The reduced expression of flagella synthesis genes has been demonstrated to result in marked swimming defect and robust biofilm production in *Salmonella* spp. [38]. Interestingly, ethanol at subinhibitory concentrations was found to repress the expression of a flagellar basal body protein (FlgF) and a flagellar assembly protein (FliH) in *S*. Enteritidis in a previous proteomics study [29]. Downregulation of FlgF and FliH might explain the reduced swimming motility and subsequent enhanced biofilm production by *S*. Enteritidis in the presence of ethanol in the current work.

### 3.4. Influence of Ethanol at Subinhibitory Concentrations on Bacterial Surface Characteristics

Bacterial surface characteristics (e.g., hydrophobicity and auto-aggregation) were tested to explore their relationship with biofilm formation in *S*. Enteritidis. As shown in Table 2, ethanol at subinhibitory concentrations did not significantly (*p* > 0.05) alter bacterial hydrophobicity. On the contrary, *S*. Enteritidis treated with ethanol displayed significantly (*p* < 0.05) greater capacity to auto-aggregate than the control group. Moreover, cell auto-aggregation was more pronounced as the concentration of ethanol increased from 2.5% to 5.0% (Table 2). These results indicate a positive correlation between biofilm formation and cell auto-aggregation in *S*. Enteritidis under ethanol stress.

Auto-aggregation is defined as cell-to-cell interactions wherein bacterial pathogens spontaneously aggregate together [39]. A higher auto-aggregation rate is an indicator of a stronger cell-to-cell interaction, which is beneficial for bacterial pathogens to produce biofilms. Xu et al. [26] observed that auto-aggregation enhanced biofilm formation of several foodborne pathogens (e.g., *S.* Typhimurium) under NaCl treatment. Therefore, cell-to-cell interactions might be a factor to favor *S*. Enteritidis biofilm formation under subinhibitory ethanol concentrations in the current work.

### 3.5. Influence of Ethanol at Subinhibitory Concentrations on the Expression of Selected Genes and sRNAs

**Figure 4:**
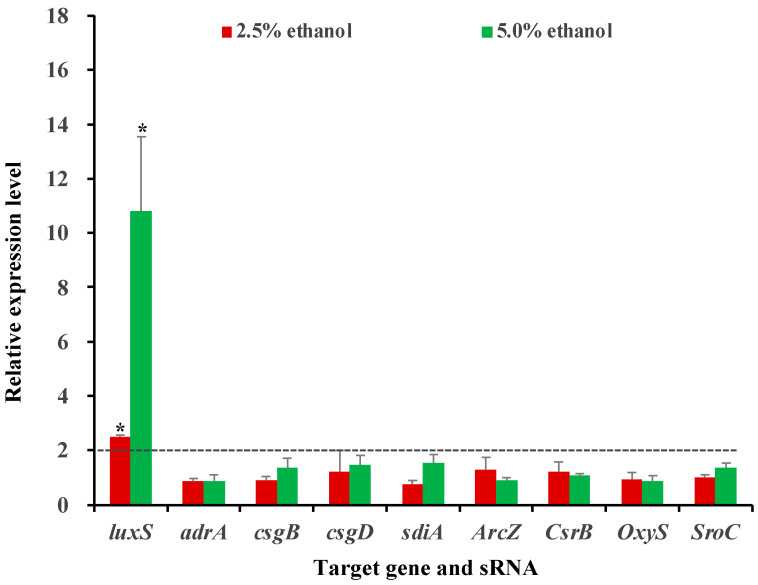
Relative expression levels of selected genes and sRNAs under subinhibitory ethanol concentrations (1/4 MIC, 2.5%; 1/2 MIC, 5.0%). Stars (*) above vertical bars represent a significant difference (*p* < 0.05) in gene expression.

The expression levels of three attachment genes (*adrA, csgB*, and *csgD*), two quorum-sensing genes (*luxS* and *sdiA*) and four sRNAs (ArcZ, CsrB, OxyS, and SroC) under ethanol stress were determined by RT-qPCR analysis in the current work. Out of these genetic elements, only the *luxS* gene was significantly (*p* < 0.05) differentially expressed after exposure to ethanol; this gene was upregulated by 2.49- and 10.08-fold under 2.5% and 5.0% ethanol stresses, respectively. On the contrary, all other tested genes and sRNAs did not show altered expression patterns under ethanol stress in *S*. Enteritidis (Figure 4).

The aforementioned genetic elements were selected for RT-qPCR test due to their key role in biofilm formation of *Salmonella* spp. under well-defined laboratory conditions favorable for bacterial growth [14,32]. However, their expression patterns may vary in response to different environmental stimuli [14,32], and it remains largely unknown which elements are involved in bacterial biofilm formation under sub-MIC levels of ethanol. In the current work, three attachment genes (*adrA, csgB*, and *csgD*) were not significantly (*p* > 0.05) differentially expressed in *S*. Enteritidis, which could be a reason for the remained cell attachment ability under ethanol stress. Similarly, it seemed that four sRNAs (ArcZ, CsrB, OxyS, and SroC) also did not participate in biofilm formation of this pathogen stimulated by ethanol, as revealed by RT-qPCR analysis. However, exposure to the alcohol resulted in the upregulation of the *luxS* gene, indicating the involvement of this gene in ethanol-induced biofilm formation in *S*. Enteritidis. In line with this observation, sub-MIC levels of plant-derived quercetin could retard biofilm formation in *S*. Enteritidis and *S.* Typhimurium, accompanied by a reduced expression of the *luxS* gene [17]. It thus seemed that the *luxS* gene might play a vital role in biofilm formation of *Salmonella* spp. under some sublethal environmental stresses.

The *luxS* gene participates in the synthesis of autoinducer-2, which acts as a universal quorum-sensing molecule in bacterial pathogens [24,40]. The LuxS quorum-sensing system offers a means for cell-to-cell communications, which is critical for bacterial biofilm production [41,42]. Deletion of the *luxS* gene has been demonstrated to significantly (*p* < 0.05) reduce biofilm production by *S.* Typhimurium in LB medium [43]. Interestingly, in the current work, ethanol at subinhibitory concentrations induced the expression of the *luxS* gene, along with enhanced biofilm production in *S*. Enteritidis. Therefore, the *luxS* gene might contribute to bacterial biofilm formation under ethanol stress by regulating quorum-sensing processes.

It is noteworthy that the stimulation of biofilm formation by ethanol has been observed in a model organism of *S*. Enteritidis in the current work. The broader impact of this phenomenon can be addressed by a population-wide survey incorporating more *S*. Enteritidis isolates in future studies. Moreover, bacterial pathogens are able to exist in multispecies biofilms in food-related environments [44]. Thus, it would also be of importance to further assess ethanol-mediated biofilm formation using a multispecies bacterial cocktail.

## 4. Conclusions

Ethanol at subinhibitory concentrations could accelerate biofilm production in *S*. Enteritidis, accompanied by reduced swimming motility and enhanced auto-aggregation ability. Moreover, the *luxS* gene located on a quorum-sensing system was upregulated in response to sub-MIC levels of ethanol, which suggested the involvement of bacterial quorum-sensing in ethanol-induced biofilm formation. These results demonstrate that alcohols should be used with caution to avoid the presence of subinhibitory ethanol concentrations which may pose a risk to food safety by triggering bacterial biofilm formation.

## Figures and Tables

**Table 1:** Primer sequences of biofilm-related genes and sRNAs.

Gene/sRNA	NCBI Accession No. or Gene ID	Sequence (5′ to 3′)	Reference
*luxS*	CAR34242.1	F: ATGCCATTATTAGATAGCTT	[14]
		R: GAGATGGTCGCGCATAAAGCCAGC	
*adrA*	CAR31954.1	F: GAAGCTCGTCGCTGGAAGTC	[15]
		R: TTCCGCTTAATTTAATGGCCG	
*csgB*	CAR33485.1	F: TCCTGGTCTTCAGTAGCGTAA	[14]
		R: TATGATGGAAGCGGATAAGAA	
*csgD*	CAR33486.1	F: TCCTGGTCTTCAGTAGCGTAA	[15]
		R: TATGATGGAAGCGGATAAGAA	
*sdiA*	CAR32642.1	F: AATATCGCTTCGTACCAC	[15]
		R: GTAGGTAAACGAGGAGCAG	
ArcZ	2847690	F: ACTGCGCCTTTGACATCATC	[15]
		R: CGAATACTGCGCCAACACCA	
CsrB	1254489	F: CAAAGTGGAAAGCGCAGGAT	[15]
		R: TGACCTTACGGCCTGTTCAT	
OxyS	6797054	F: TAACCCTTGAAGACACCGCC	[15]
		R: ACCAGAGGTCCGCAAAAGTT	
SroC	6793706	F: GGGACTCCTGTCCTCTCGAT	[15]
		R: CAGCGCTACCCTCGAAGATT	
16S rRNA	X80676.1	F: AGGCCTTCGGGTTGTAAAGT	[15]
		R: GTTAGCCGGTGCTTCTTCTG	

**Table 2:** Influence of subinhibitory concentrations of ethanol (1/4 MIC, 2.5%; 1/2 MIC, 5.0%) on cell surface characteristics of *S*. Enteritidis.

Properties	0.0% Ethanol	2.5% Ethanol	5.0% Ethanol
Hydrophobicity (%)	3.92 ± 1.33 ^a^	4.98 ± 0.65 ^a^	3.60 ± 1.16 ^a^
Auto-aggregation (%)	40.53 ± 1.35 ^c^	58.92 ± 1.06 ^b^	72.32 ± 0.45 ^a^

Note: Different letters in a row represent a significant difference (*p* < 0.05).

## Data Availability

All data are available in the article.
